# Governing the misconduct of OTA platforms: A tripartite evolutionary game analysis considering the collaborative supervision of airlines and consumers

**DOI:** 10.1371/journal.pone.0305876

**Published:** 2024-08-22

**Authors:** Wenjian Li, Jiwen Tai, Jingxuan Zhou, Liya Ba, Gang Xie

**Affiliations:** 1 School of Management, Jiangsu University, Zhenjiang, Jiangsu, China; 2 School of Flight Technology, Civil Aviation Flight University of China, Deyang, Sichuan, China; 3 School of Foreign Languages, Civil Aviation Flight University of China, Deyang, Sichuan, China; University of Palermo, ITALY

## Abstract

Online travel agency (OTA) platforms frequently engage in unfair behaviors that infringe on the legitimate rights and interests of consumers and airlines in the ticket sale market. Effective governance of the OTA platforms’ misconduct has become an urgent topic. In order to address the governance dilemma of OTA platforms’ misconduct, a tripartite evolutionary game model considering the collaborative supervision between airlines and consumers is constructed. This study analyzes the evolutionary path and stable strategy of the three participants, airlines, platforms and consumers by numerical simulation. The results show that some actions, such as airlines’ strict control of ticket sales resources and high fines on the platform, reducing the cost of customers’ rights protection, and effectively guiding online public opinion, can benefit airlines and consumers and enhance their willingness to cooperate in supervision. Legitimate consumer rights protection not only brings negative public opinion and image loss to airlines, but also to platforms, which can force airlines to impose stricter constraints on platforms and force platforms to strengthen self-restraint. Therefore, a market mechanism instead of government regulatory that can effectively suppress platforms misconduct should be established to promote platforms self-regulation through a collaborative effort between airlines and consumers. Some special measures that guide the interests of three participators are also provided.

## Introduction

Online travel agency (OTA) platforms provide online travel-related services that include answering online inquiries and enabling ticket purchases [1]. As an “intermediary,” the OTA platform acts a channel for online ticket sales that connects airlines and consumers to enable efficient and convenient ticketing [2]. Network effects have allowed the OTA platform economy to trend toward high market concentration. For example, in 2020, the market share of Ctrip platforms in China reached 58.2%, demonstrating high-concentration characteristics [3]. Following the concentration trend, the market misconduct by OTA platforms, rooted in the platform’s dual advantages in both technology and traffic, has become increasingly prevalent [4]. Unfair competition, such as big data-enabled price discrimination, bundle sales, cross-boundary selling, or other offenses, including deceptive marketing through low-cost tickets, leakage of consumer privacy, and illegal ticket refund and modification, are especially common [5–7]. An famous example of such misconduct was reported in the Chinese *People’s Daily* in 2020 [8]: A customer named Ms. Zhou bought an air ticket at a price several hundred yuan higher than that of her friends, who bought tickets for the same flight on the same day through an OTA platform. The price she paid was nearly 1,000 yuan higher than when she searched for a flight. As discriminatory pricing severely violates consumers’ legitimate rights and interests, OTA platforms are always passing the buck—or the burden—to airlines [9]. They usually shirk their responsibility on the grounds that airlines have not provided processing policies.

The literature dealing with the factors of price dispersion in the airline industry reports a correlation between competition and price discrimination. Some OTAs’ features related to the presence of airline competition, such as the ability to display and combine products of different airlines, lead to higher price dispersion in this channel [10]. The behavior of online sales platforms charging different prices to different customers is often considered unfair and to be detrimental to consumers [11]. The increasing platform misconduct has exposed a crisis of trust in the airline ticket service market. It not only has harmed the interests of airlines and consumers, but also seriously disrupted the market order of air ticket services, ultimately hindering the healthy development of the platform economy [12]. Recently, whether to use the official website for sales or OTA services has become a decision-making problem for airlines. Researchers have indicated that airlines should reduce the use of OTA platforms if they have a large loyal consumer base or if OTA platforms are highly competitive [13]. For the low-cost carriers, the main channel for selling tickets should be the OTA platform [14, 15]. However, in the era of platform economy, it is unrealistic for even high cost airlines to completely abandon OTAs channels. In order to minimize the negative impact of proxy platforms as much as possible, airlines try to regulate OTAs behaviors. For example, three airlines in china, such as Southern Airlines, Shenzhen Airlines, and Hainan Airlines, had worked together to put pressure on the platform to restrain their own violations, using a reduction of business as a threat [16]. As the misconduct from many service platforms, including OTAs, harms the economy and society [12], governance of market misconduct on OTA platforms has also become a pressing issue and received considerable social attention. It is worthwhile to determine whether there is a reasonable mechanism that can effectively prevent platforms taking misconduct.

Scholars have conducted many studies on the governance of unfair market behaviors from these online service platforms. There is considerable attention paid here to the governance of platforms unfair behavior enabled by new technologies. Guo et al. called for tackling unfair competition of platform enterprises to cope with the challenges of emerging technologies and rapid changes in the economic environment [17]. Cheng and Cheng argued that information alienation should be suppressed through the support from different levels, such as the user, media, and government [18]. Miao et al. discussed the governance approach of subversive technological dissimilation from the perspectives of technology, organization management, and social system by taking “deepfake” technology as an example [19]. Chung et al. found that AI technology can be used on social media to prevent misconduct, such as privacy disclosure [20]. Recently, many service platforms have adopted discriminatory pricing based on user behavior, using big data technologies [21]. Scholars have focused on the governance of discriminatory pricing behavior in the context of big data to shed light on the governance mechanism behind this phenomenon [22]. The difficulty in safeguarding the interests of consumers is not easy to be solved, considering the powerful energy of the platform and the algorithms opacity in the context of new technology. It is necessary for multi relevant subject to participate in the governance of platform violations [23].

Evolutionary game model and simulation method have used to analyze the mechanisms of two-party or three-party supervision, for the cooperation of multi party can effectively safeguard the legitimate rights and interests of consumers. Liu et al., for example, built tripartite evolutionary game model of the service platforms, government, and consumers to explain how to regulate discriminatory pricing behavior under big data background in the service [22]. Their model considered the risk aversion of service platforms and results showed when the service platform is risk-averse, the government can use tax rates to drive platform compliance operations, or the government can choose strict measures, such as high penalties, to supervise the platform for restraining discriminatory pricing. Xing et al. built a two-party evolutionary game model between the platform and consumers to investigate the role of the data portability rights and the impact of data value on the consumers exercise of rights to data portability [24]. They suggested introducing data portability rights to curb big data discriminatory pricing behavior. Xu and Li proposed a collaborative supervision mechanism between the government and a third party using an evolutionary game model of large platforms, small platforms, and the government. Their study showed that active small platforms supervision could alleviate the shortage of government supervision and effectively regulate big data discriminatory pricing behavior of big service platforms [25]. Lei et al. analyzed evolutionary game strategies between the platforms and customers in online car-hailing service considering the mechanism of coordinated supervision, such as the punishment of government and consumers’ fairness concern, and found that the combination of government punishment and reputation loss brought about by consumer supervision can force the platform to adopt fair pricing [26]. Fu et al. established an evolutionary game model between the online car-hailing platform and the government regulators considering the influence of media to solve the moral risk of online car-hailing. The results showed that media coverage affects the strategic choice of online carhailing platform and regulators and media supervision played an important role in supplement to government supervision [23]. Peng et al. study the behavior of the government, platforms, drivers and passengers to regulate online car-hailing market by an evolutionary game model of four parties. The simulation showed that increasing the rewards and punishments increase help to strengthen the self-restraint ability of the platform and the drivers, and reducing managerial cost can accelerate the system to the stable state [27]. Zuo et al. constructs evolutionary games model of online ride-hailing platforms, drivers, and passengers under different government regulations regarding penalty policy, incentive policy, policy adaptability, and public participation. They found government regulations help stabilize the system, and platforms as agents of government regulations need to take responsibility for implementing incentive policy, improving policy adaptability, and rewarding public participation [28].

Scholars are in consensus on the urgent for governance of service platforms indulging in unfair competition or other illegal activities. However, at present, there is little literature that analyzes the game strategies between airlines, OTA platforms, and consumers considering the collaborative mechanism for the governance of the misconduct behaviors of OTA platforms. Current researches have given more attention to the government supervision of vary service performs, focusing on the governance mechanisms and measures based on collaborative supervision between the government and consumers. Although government regulations and supervision are of great importance, further exploration of some mechanisms is also necessary to cope with the regulation dilemma of the OTA platforms misconduct, such as initiatives of airlines and ways to promote self-regulation of OTA platforms through a synergistic relationship between airlines and consumers. A specific interest–game relationship exists in the OTA market. Consumers are the end users of OTA platforms and also direct victims of misconduct. In theory, when a consumer’s rights are violated, the consumer can file a complaint. However, the victim may give up the right protection owing to its interminable process and high cost [29]. Obviously, a regulation that relies solely on the supervision of the consumer is not reliable, and airlines must participate in the regulation of platform behaviors. There are two main types of airlines: low-cost carriers (LCCs) and non-low-cost carriers (non-LCCs). In general, non-LCCs have a higher degree of control over the distribution of air tickets than LCCs, making them a key regulator governing OTA platforms and helping consumers with the right protection. For example, some airlines inhibit unfair competition of platforms in the ticket agency market by increasing direct sales channels and developing frequent flyer programs [13]; they ask OTA platforms to rectify this by not displaying the markup service on the search list page and product list page of the platform’s main process. The misconduct of OTA platforms is directly related to the number of terminal users and sales traffic advantages. For example, Ctrip platform has a large penetration rate in the middle value-end and high value-end consumers of ticket demand market, and the number of two types of consumers accounts for nearly half. Ctrip platform have controlled the needs of more high-quality customer, thus increasing airlines’ dependence on the platforms.If airlines are more dependent on the number of users and the traffic advantages owned by the platforms, they will weaken their control over OTA platforms [30]. Some LCCs find it difficult to control platforms because of their excessive dependence on them [14]. The game between airlines and platforms has a love-and-kill relationship. However, fortunately, in the Internet era, public opinion on the internet has spread rapidly and creates a strong pressure [31]. Public opinion generated by consumers on the Internet can trigger public concerns about the fairness of platforms and airlines, which then could affect the image and reputation of them [32], forcing non- LCCs that have the ability to constrain the platforms to strengthen their regulatory responsibilities. Ultimately, it could force the platforms to exercise greater self-restraint [33]. Therefore, the governance of platform misconduct requires a collaboration of consumer power and airline regulation.

Based on these contexts, and owing to the weak control of LCC on the platform, only non-LCCs are considered as the regulatory entities. This study constructs an evolutionary game model of non-LCCs, OTA platforms, and consumers, considering some factors related to collaborative mechanisms such as the degree of airlines’ control over air ticket selling, penalties for the OTA platforms, airlines’ assistance to consumer rights protection, and online public opinion pressure generated by consumers. Numerical simulation analysis is used to clarify the beneficial evolution and stability strategy of three party game. Our model adds a knowledge on how airlines and consumers can work together to promote the self-regulation mechanism of OTA platforms. In our study, collaborative governance methods to curb the misconduct of OTA platforms provide a theoretical basis for the practical improvement of the governance and supervision system of platforms misconduct.

## Problem description and model construction

### Theoretical basis and the game problem

Evolutionary game is a combination of game theory and evolution analysis of the players’ behavior [22]. The evolutionary game theory was first applied to the field of population biology for addressing the dynamic strategy selection problems [22]. Common forms are two-party or tripartite evolutionary game model. Later, it was introduced into socioeconomic systems and provided a new analytical approach for the co-evolutionary study of the participators’ behavior [34]. The evolutionary game theory is effective tool to explain the complex game relationships and strategies’ evolution. The game relationship among three parties on the service platform is in a dynamical change, requiring time-related model and methods to clarify its law. Based on evolutionary game theory and numerical simulation, the modeling of airlines, platforms and customers can clarify the players’ behavior dynamics and stability strategy for the collaborative supervisory purposes [35]. In this paper, the collaborative supervision of airlines and customers has a practical significance to curb unfair behavior of platforms.

Airlines, OTA platforms, and consumers are the three subjects in the online ticket service market. Airlines curb and regulate online ticketing on OTA platforms through their control over air ticket sales. They will punish OTA platforms for misconduct harming the interests of airlines and consumers. As a channel for online ticket sales connecting the airline and the consumer, OTA platforms should be devoted to air ticket services and transactions. However, to maximize their own interests, the OTA platforms can take advantage of their traffic advantages and technical capabilities to carry out misconduct such as big data discriminatory pricing [36], privacy leakage [37], and bundle sales [38], which seriously disrupt the order in the online air ticket sales market.

Compared with dominant OTA platforms, consumers are often relatively weak, and their reporting of platform behaviors is subject to high evidentiary costs. Thus, their enthusiasm for participating in the platform supervision is low. It is often difficult to rely on consumers alone to achieve misconduct governance of OTA platforms, and it is necessary to cooperate with airlines to govern the misconduct of OTA platforms.

### Basic assumptions

Hypothesis 1. Making the set of participants {1, 2, 3}, there are three groups of participants in the model, namely, the airlines, OTA platforms and consumers. In this set, the airline is Participant 1, OTA platform is Participant 2, and consumer is Participant 3. All of these are bounded rationality decision-makers.

Hypothesis 2. The strategy set of airlines is {positive regulation, negative regulation}. Positive regulation refers to airlines’ active supervision—for example, monitoring whether the platform provides price-added products and services through packages and labels, conducting bundling sales, and implementing price discrimination. Positive airlines will also put forward strict prices and product display specifications on OTA platforms and take severe regulation measures such as fining OTA platforms’ misconduct, according to the investigation results. In addition, positive airlines will provide a positive response to consumers rights protection, and promptly verify and decide whether to punish the platform based on the verification results. “Negative regulation” means that the airlines are a mere formality when performing their supervisory duties on OTA platforms, do not severely punish the platforms, respond perfunctorily to consumers’ rights protection, and do not strongly demand the platforms to rectify. The strategy set for OTA platforms is {complaint operation, misconduct}. “Misconduct” means that OTA platforms act with disregard for the airlines and consumers to expand their own interests to the largest degree, including big data discriminatory pricing, privacy infringement, bundling sale, bug-sell, deceptive low-cost tickets and illegal ticket refund. A complaint operation refers to a situation in which OTA platforms follow the fair-trade of the market, fulfill their social responsibility, and avoid misconduct. Consumers strategy set is {rights protection, non-rights protection}. “Rights protection” refers to the strategy that consumers use to protect their rights and interests through some tools, like the social media. They share their unfortunate experiences on Weibo and WeChat. Their stories are also reported in online media and spread across social networks. Consequently, platforms or airlines gain negative word-of-mouth, which reduces both entities’ customer base. Consumers complain about the hotline, and the results are published online and spread. Once their stories and processing results are widely considered by the public and become a popular topic, public opinion is formed. The negative impact of public opinion on the reputation and image of airlines and platforms is amplified many times. “Non-rights protection” means that the consumers are reluctant to take measures to safeguard their rights and interests. There are eight game strategy combinations among airlines, OTA platforms, and consumers, as shown in Table 1.

**Table 1:** Eight game strategy combinations.

Serial Number	Strategy Combination
1	(positive regulation, complaint operation, rights protection)
2	(positive regulation, complaint operation, non-rights protection)
3	(positive regulation, misconduct, rights protection),
4	(positive regulation, misconduct, non-rights protection)
5	(negative regulation, complaint operation, rights protection)
6	(negative regulation, complaint operation, non-rights protection)
7	(negative regulation, misconduct, rights protection)
8	(negative regulation, misconduct, non-rights protection)

Hypothesis 3. The probability that airlines choose “positive regulation” strategy is *x* and the probability of choosing “negative regulation” strategy is 1*-x*. The probability of OTA platforms choosing “complaint operation” strategy is *y*, and the probability of choosing “misconduct” is 1*-y*. The probability of consumers choosing “rights protection” strategy is *z*, and the probability of choosing “non-rights protection” is 1*-z*. Among them, 0≤*x*≤1, 0≤*y*≤1, and 0≤*z*≤1.

Hypothesis 4. When airlines actively control platforms, the increased cost is *C*_*1*_ and the increased income is *R*_*1*_. When platforms misbehave, the direct economic loss to airlines is *L*_*1*_. For example, the platforms charge a high refund fee, but blame the airlines; this is a negative consumer experience that reduces continuous consumption. When the airlines actively control the platforms for misconduct and consumers protect their rights, the airlines will impose a fine of *ηλF* on the platforms, where *η* is the control degree of ticket sales resources of airlines, *λ* is the influence of consumers’ rights protection on online public opinion, and *F* is the upper limit of the airlines’ fine. When the airlines negatively control the platforms and consumers defend their rights, the airlines will experience reputation loss *λK*, where *λ* is the degree of influence of online public opinion caused by consumers rights protection and *K* is the base of the airlines’ reputation loss. When the airlines passively control non-compliant platforms and consumers do not protect their rights, there will exist a potential loss of *H* owing to unknown hidden dangers. For example, if airlines have negative controls and consumers do not safeguard their rights, platform misconduct cannot be discovered and stopped by the airlines in time, which will result in unpredictable losses to the airlines. For example, consumers affect the consumption of other people they know through interpersonal contact. Consequently, the airlines’ customer base takes an invisible hit, and the airlines must spend more energy maintaining customer loyalty.

Hypothesis 5. When platforms comply, the increased operating cost is *C*_*2*_. When platforms’ behavior is out of compliance, the increased revenue is *R*_*2*_. When the platforms’ behavior is out of compliance and consumers actively protect their rights, platforms will experience reputation loss *λV*, where *V* is the basis of platforms’ reputation loss. If consumers defend their rights, but after active control by the airlines, it is found that the platforms’ behavior is compliant, they will gain reputation revenue *λQ* owing to the consolidation of positive image. *Q* is the basis of platforms’ reputational revenue. When consumers defend their rights, and if the airlines respond negatively, it will bring reputation loss *λV* to the platforms because the airlines do not actively organize investigations and fail to verify the authenticity of infringement in time, resulting in negative public opinion toward the platforms without misconduct. To maintain their reputation, the platforms will take reputation protection measures such as marketing actions to clarify the facts and invite third-party institutions to verify their innocence to the outside world, thus paying reputation protection cost *λM*. Simultaneously, through the implementation of reputation protection measures, platforms will regain the trust of the public and receive reputation protection revenue *λN*. *M* is the basis of reputation protection costs and *N* is the basis of reputation protection revenue.

Hypothesis 6. The cost of consumers rights protection is *C*_*3*_. Airlines will comfort consumers’ rights protection through *η*^*2*^*λ*^*2*^*W*, where *W* is the basis of the comfort. When platforms engage in misconduct, the loss of consumers is *L*_*3*_. When airlines actively control platforms engaged in misconduct and consumers protect their rights, the platforms will compensate consumers for *m* times of loss, that is *mL*_*3*_, and *m* is the compensation coefficient. When the airlines actively control the platforms engaged in misconduct, to encourage consumers to participate in governance, the consumers who act to protect their rights will receive a certain proportion *α* of transfer of the fine revenue of airlines on platforms, which is *αηλF*, and *α* is the transfer ratio, 0≤*α*≤1.

The model parameters and their definitions are listed in Table 2.

**Table 2:** Parameters and meanings.

*Parameters*	*Meanings*	*Range*
*x*	The probability of positive regulation by airlines	[0,1]
*y*	The probability of non-misconduct by platforms	[0,1]
*z*	The probability of consumers rights protection	[0,1]
*C*_*1*_	Increase in cost caused by the positive regulation of airlines	
*R*_*1*_	Increase in revenue caused by the positive regulation of airlines	
*L*_*1*_	Airlines’ economic loss caused by platform misconduct	
*η*	The control degree of airlines on air ticket sales resources	[0,1]
*λ*	Degree of the influence of online public opinion caused by rights protection	[0,1]
*F*	The upper limit of fine when airlines actively regulate platforms	
*K*	The basis of the reputation loss of airlines	
*H*	Airlines’ potential loss caused by hidden dangers	
*C*_*2*_	Increase in operating cost when platforms are compliant	
*R*_*2*_	Increase in revenue when there is platform misconduct	
*V*	The basic quantity of platforms’ reputation loss	
*Q*	The basic quantity of platforms’ reputation revenue	
*M*	The basic quantity of platforms’ reputation protection cost	
*N*	The basic quantity of platforms’ reputation protection revenue	
*C*_*3*_	The rights protection cost of consumers	
*W*	The basic quantity of the comfort of consumers obtained by rights protection	
*L*_*3*_	The loss of consumers caused by platform misconduct	
*m*	Coefficient of platforms’ compensation for the consumers’ loss	
*α*	Transfer proportion of the fine obtained by consumers for rights protection	[0,1]

According to the above parameters in the model, we obtain the game revenue matrix for the airline, OTA platform, and consumer, as shown in Table 3.

**Table 3:** The revenue matrix of three-party game.

airline and consumer		There is platform misconduct (1-*y)*	There is not platform misconduct (*y)*
The airline’s regulation is negative (1-*x*)	The consumer protects the rights (*z*)	$-L_{1}-\lambda K-\eta \lambda^{2}W$ *R*_2_−*λV* $-L_{3}-C_{3}+\eta^{2}\lambda^{2}W$	$-\lambda K-\eta^{2}\lambda^{2}W$ $-C_{2}-\lambda V-\lambda M+\lambda N$ $-C_{3}+\eta^{2}\lambda^{2}W$
	The consumer doesn’t protect the rights (*1-z*)	−*L*_1_−*H* *R*_2_ −*L*_3_	0 −*C*_2_ 0
The airline’s regulation is positive (*x*)	The consumer protects the rights (*z*)	$-L_{1}-C_{1}+R_{1}+\eta \lambda F-\alpha \eta \lambda F-\eta^{2}\lambda^{2}W$ $R_{2}-\eta \lambda F-mL_{3}-\lambda V$ $-L_{3}-C_{3}+mL_{3}+\alpha \eta \lambda F+\eta^{2}\lambda^{2}W$	$-C_{1}+R_{1}-\eta^{2}\lambda^{2}W$ $-C_{2}+\lambda Q$ $-C_{3}+\eta^{2}\lambda^{2}W$
	The consumer doesn’t protect the rights (1-*z*)	−*L*_1_−*C*_1_+*R*_1_ *R*_2_ −*L*_3_	−*C*_1_+*R*_1_ −*C*_2_ 0

Note: The first to third rows of the revenue matrix are revenue of the airline, the OTA platform, and the consumer respectively.

### Model construction

Suppose the expected income of the airlines from choosing a positive regulation strategy is *E*_*11*_, the expected revenue from choosing a negative regulation strategy is *E*_*12*_, and the average expected revenue is *E*_*1*_. Then,

$$\begin{array}{l} E_{11}=(1-y)z(-L_{1}-C_{1}+R_{1}+\eta \lambda F-\alpha \eta \lambda F-\eta^{2}\lambda^{2}W)+(1-y)(1-z)\\ \left(-L_{1}-C_{1}+R_{1})+yz(-C_{1}+R_{1}-\eta^{2}\lambda^{2}W)+y(1-z)(-C_{1}+R_{1}\right) \end{array}$$ (1)

$$E_{12}=(1-y)z(-L_{1}-\lambda K-\eta^{2}\lambda^{2}W)+(1-y)(1-z)(-L_{1}-L_{3})+yz(-\lambda K-\eta^{2}\lambda^{2}W)$$ (2)

$$E_{1}=xE_{11}+(1-x)E_{12}$$ (3)

According to the Malthusian dynamic equation [39], let *t* be the evolution time. Then, the replicated dynamic equation of the airlines adopting the positive regulation strategy is

$$\begin{array}{l} F(x)=\frac{dx}{dt}=x(E_{11}-E_{1})=x(1-x)((-H)y+((1-\alpha )\eta \lambda F+\lambda K-H)z\\ +(H-(1-\alpha )\eta \lambda F)yz+R_{1}-C_{1}+H) \end{array}$$ (4)

Suppose the expected revenue of OTA platforms when choosing the non-misconducting strategy is *E*_*21*_, the expected revenue from choosing the misconducting strategy is *E*_*22*_, and the average expected revenue is *E*_*2*_. Then,

$$E_{21}=(1-x)z(-C_{2}-\lambda V-\lambda M+\lambda N)+(1-x)(1-z)(-C_{2})+xz(-C_{2}+\lambda Q)+x(1-z)(-C_{2})$$ (5)

$$E_{22}=(1-x)z(R_{2}-\lambda V)+(1-x)(1-z)R_{2}+xz(R_{2}-\eta \lambda F-mL_{3}-\lambda V)+x(1-z)R_{2}$$ (6)

$$E_{2}=yE_{21}+(1-y)E_{22}$$ (7)

Then, the replicated dynamic equation of OTA platforms adopting the non-misconducting strategy is

$$\begin{array}{l} F(y)=\frac{dy}{dt}=y(E_{21}-E_{2})=y(1-y)((-\lambda M+\lambda N)z\\ +(\eta \lambda F+mL_{3}+\lambda Q+\lambda V+\lambda M-\lambda N)xz-C_{2}-R_{2}) \end{array}$$ (8)

Suppose the expected revenue of consumers choosing a rights protection strategy is *E*_*31*_, the expected revenue from choosing a non-rights protection strategy is *E*_*32*_, and the average expected revenue is *E*_*3*_. Then,

$$\begin{array}{l} E_{31}=(1-x)(1-y)(-L_{3}-C_{3}+\eta^{2}\lambda^{2}W)+(1-x)y(-C_{3}+\eta^{2}\lambda^{2}W)\\ +x(1-y)(-L_{3}-C_{3}+\alpha \eta \lambda F+\eta^{2}\lambda^{2}W+mL_{3})+xy(\eta^{2}\lambda^{2}W-C_{3}) \end{array}$$ (9)

$$E_{32}=(1-x)(1-y)(-L_{3})+x(1-y)(-L_{3})$$ (10)

$$E_{3}=zE_{31}+(1-z)E_{32}$$ (11)

Then, the replicated dynamic equation of the consumers’ rights protection strategy is

$$F(z)=\frac{dz}{dt}=z(E_{31}-E_{3})=z(1-z)((\alpha \eta \lambda F+mL_{3})x-(\alpha \eta \lambda F+mL_{3})xy-C_{3}+\eta^{2}\lambda^{2}W)$$ (12)

### Stability analysis of evolutionary strategy under the joint action of three parties

#### Replicated dynamic system and Jacobian matrix

Combining Eqs (4), (8), and (12), the replicated dynamic system of the airlines, OTA platforms, and consumers is obtained as follows:

$$\left\{ \begin{array}{l} F(x)=x(1-x)((-H)y+((1-\alpha )\eta \lambda F+\lambda K-H)z+(H-(1-\alpha )\eta \lambda F)yz+R_{1}-C_{1}+H)\\ F(y)=y(1-y)((-\lambda M+\lambda N)z+(\eta \lambda F+mL_{3}+\lambda Q+\lambda V+\lambda M-\lambda N)xz-C_{2}-R_{2})\\ F(z)=z(1-z)((\alpha \eta \lambda F+mL_{3})x-(\alpha \eta \lambda F+mL_{3})xy-C_{3}+\eta^{2}\lambda^{2}W) \end{array}\right.$$ (13)

According to the method proposed by Friedman, the evolutionary stability strategy (ESS) can be obtained from a local stability analysis of the Jacobian matrix of the system. The Jacobian matrix of the replicated dynamic system can be obtained from Eq (13) as follows [40]:

$$J=[\begin{array}{ccc} F_{1}(x) & F_{2}(x) & F_{3}(x)\\ F_{1}(y) & F_{2}(y) & F_{3}(y)\\ F_{1}(z) & F_{2}(z) & F_{3}(z) \end{array}]$$

Among them,

$$F_{1}(x)=(1-2x)((-H)y+((1-\alpha )\eta \lambda F+\lambda K-H)z+(H-(1-\alpha )\eta \lambda F)yz+R_{1}-C_{1}+H)$$

$$F_{2}(x)=x(1-x)(-H+(H-(1-\alpha )\eta \lambda F)z)$$

$$F_{3}(x)=x(1-x)(((1-\alpha )\eta \lambda F+\lambda K-H)+(H-(1-\alpha )\eta \lambda F)y)$$

$$F_{1}(y)=y(1-y)((\eta \lambda F+mL_{3}+\lambda Q+\lambda V+\lambda M-\lambda N)z)$$

$$F_{2}(y)=(1-2y)((-\lambda M+\lambda N)z+(\eta \lambda F+mL_{3}+\lambda Q+\lambda V+\lambda M-\lambda N)xz-C_{2}-R_{2})$$

$$F_{3}(y)=y(1-y)((-\lambda M+\lambda N)+(\eta \lambda F+mL_{3}+\lambda Q+\lambda V+\lambda M-\lambda N)x)$$

$$F_{1}(z)=z(1-z)((\alpha \eta \lambda F+mL_{3})-(\alpha \eta \lambda F+mL_{3})y)$$

$$F_{2}(z)=z(1-z)(-(\alpha \eta \lambda F+mL_{3})x)$$

$$F_{3}(z)=(1-2z)((\alpha \eta \lambda F+mL_{3})x-(\alpha \eta \lambda F+mL_{3})xy-C_{3}+\eta^{2}\lambda^{2}W)$$

According to Eq (13), eight local equilibrium points are obtained:

$$E_{1}(0,0,0),\;E_{2}(0,0,1),\;E_{3}(0,1,0),\;E_{4}(0,1,1),\;E_{5}(1,0,0),\;E_{6}(1,0,1),\;E_{7}(1,1,0),\;E_{8}(1,1,1).$$

According to Lyapunov’s first method, when all the eigenvalues of the Jacobian matrix are negative, the equilibrium point is a stable point.

### Stability analysis of equilibrium point

Let the corresponding Jacobian matrix of *E*_1_(0,0,0) be *J*_*1*_. Then,

$$J_{1}=[\begin{array}{ccc} \lambda_{1} & 0 & 0\\ 0 & \lambda_{2} & 0\\ 0 & 0 & \lambda_{3} \end{array}]$$

The eigenvalues of *J*_*1*_ are *R*_1_−*C*_1_+*H*, −*C*_2_−*R*_2_ and *n*^2^*λ*^2^*W*−*C*_3_, respectively. Similarly, the eigenvalues of the Jacobian matrices corresponding to the other seven equilibrium points can be obtained. The calculation results are listed in Table 4.

**Table 4:** The eigenvalues of the Jacobian matrix.

Equilibrium Points	Eigenvalue *δ*_*1*_	Eigenvalue *δ*_*2*_	Eigenvalue *δ*_*3*_
***E***_**1**_ **(0,0,0)**	$R_{1}-C_{1}+H$	$-C_{2}-R_{2}$	$\eta^{2}\lambda^{2}W-C_{3}$
***E***_**2**_ **(0,0,1)**	$(1-\alpha )\eta \lambda F+\lambda K+R_{1}-C_{1}$	$\lambda N-\lambda M-C_{2}-R_{2}$	$-(\eta^{2}\lambda^{2}W-C_{3})$
***E***_**3**_ **(0,1,0)**	$R_{1}-C_{1}$	$C_{2}+R_{2}$	$\eta^{2}\lambda^{2}W-C_{3}$
***E***_**4**_ **(0,1,1)**	$R_{1}-C_{1}+\lambda K$	$-(\lambda N-\lambda M-C_{2}-R_{2})$	$-(\eta^{2}\lambda^{2}W-C_{3})$
***E***_**5**_ **(1,0,0)**	$-(R_{1}-C_{1}+H)$	$-C_{2}-R_{2}$	$\alpha \eta \lambda F+mL_{3}-C_{3}+\eta^{2}\lambda^{2}W$
***E***_**6**_ **(1,0,1)**	$-((1-\alpha )\eta \lambda F+\lambda K+R_{1}-C_{1})$	$\eta \lambda F+mL_{3}+\lambda Q+\lambda V-C_{2}-R_{2}$	$-(\alpha \eta \lambda F+mL_{3}-C_{3}+\eta^{2}\lambda^{2}W)$
***E***_**7**_ **(1,1,0)**	$-(R_{1}-C_{1})$	$C_{2}+R_{2}$	$\eta^{2}\lambda^{2}W-C_{3}$
***E***_**8**_ **(1,1,1)**	$-(R_{1}-C_{1}+\lambda K)$	$-(\eta \lambda F+mL_{3}+\lambda Q+\lambda V-C_{2}-R_{2})$	$-(\eta^{2}\lambda^{2}W-C_{3})$

The following three situations are considered to discuss the system ESS, and the following proposition is established:

Proposition: When$-C_{3}+mL_{3}+\alpha \eta \lambda F+\eta^{2}\lambda^{2}W<0$, *E*_5_(1,0,0) is the only asymptotically stable point of the system; when $(\lambda Q-C_{2})-(R_{2}-\eta \lambda F-mL_{3}-\lambda V)<0$ and $-C_{3}+mL_{3}+\alpha \eta \lambda F+\eta^{2}\lambda^{2}W>0$, *E*_6_(1,0,1) is the only asymptotically stable point of the system; when $(\lambda Q-C_{2})-(R_{2}-\eta \lambda F-mL_{3}-\lambda V)>0$ and $-C_{3}+\eta^{2}\lambda^{2}W>0$, *E*_8_(1,1,1) is the only asymptotically stable point of the system.

Proof: For Case A, based on the eigenvalue expression of the equilibrium point- *E*_5_(1,0,0), the eigenvalue is *δ*_3_<0. Besides, *δ*_1_<0, *δ*_2_<0 can be inferred by *C*_2_>0, *R*_2_>0, and *R*_1_+*H*>*C*_1_. Under the conditions of Case A, not all the eigenvalues of the other seven equilibrium points are negative. Therefore, *E*_5_(1,0,0) is the only asymptotic stable point of the system. The demonstrations of asymptotic stable points in Cases B and C are similar to the proof for Case A and will not be repeated again, Q.E.D.

The features of the three cases are listed in Table 5.

**Table 5:** Features of cases.

	$-C_{3}+mL_{3}+\alpha \eta \lambda F+\eta^{2}\lambda^{2}W$	$\left(\lambda Q-C_{2})-(R_{2}-\eta \lambda F-mL_{3}-\lambda V\right)$	$-C_{3}+\eta^{2}\lambda^{2}W$
Case A *E*_5_(1,0,0)	negative	/	/
Case B *E*_6_(1,0,1)	positive	negative	/
Case C *E*_8_(1,1,1)	/	positive	positive

In Case A, when the airlines regulate positively, the platforms engage in misconduct, and if the consumers’ rights protection cost is more than the sum of their fines transferred from the airlines, comfort gain from the airlines, and compensation from the platforms, the ESS of the system is (positive regulation, misconduct, non-rights protection). In this scenario, consumers’ decisions on strategy selection are mainly influenced by the fine transferred passenger comfort gain and compensation for the loss. The lower the total benefits to customers, including the fine transferred, passenger compensation gain, and compensation for the loss, the more likely it is that the system will reach the evolutionary equilibrium (i.e., positive regulation, misconduct, non-rights protection).

In Case B, when the airlines positively regulate, the consumers safeguard their rights, and if the platforms’ net incomes from their misconduct, that is, the positive difference between the platforms’ incremental gains obtained and the total losses (including fines, compensation expenditure, reputational damage) is greater than the net earnings obtained from compliant operations; simultaneously, if the sum of fine transferred, passenger comfort gain, and compensation for the loss that the consumers obtain is greater than the cost of rights protection, the ESS of the system is (positive regulation, misconduct, rights protection). Compared with Case A, the platforms’ gains from misconduct in Case B are greater than the gains from compliant operations, causing the platforms to continue the misconduct. However, consumers’ total benefits from rights protection are greater than the costs of rights protection. Therefore, consumers tend to safeguard their rights.

In Case C, when customers’ comfort gains by rights protection are greater than its cost, and if the positive difference between the platforms’ incremental gains and the total losses from misconduct is smaller than the net earnings, that is, the difference between reputational gains and increased operating costs obtained for compliant operations, the ESS of the system is (positive regulation, compliant operation, rights protection). Under this scenario, the constraints of platform misconduct are contrary to those of Case B, which is conducive to evolving toward the equilibrium of compliant operations. In Case C, the overall benefits of consumers rights protection are somewhat reduced compared with Case B. However, if consumers are sufficiently appeased to offset the costs of pursuing their rights, they tend to protect their rights. In this situation, consumers’ strategic choices hinge mainly on the comfort gains offered by airlines and the cost of asserting their rights. An airline’s strategic choices primarily depend on its control over flight resources, fines, and online public opinion. The platforms’ strategy choices primarily hinge on the increased relative benefits of misconduct compared with compliance, compensation for consumer loss, and online public sentiment.

## Simulation analysis

According to the analysis in section 3.2, Case C (positive regulation, compliant operation, and rights protection) is the strategic choice of the three-party main. We use software MATALAB to conduct numerical simulation. Through numerical simulation analysis, the evolution paths and stable states of the three parties under the constraint of Case C are displayed more intuitively. The influence of the initial strategy selection probability of airlines, OTA platforms, and consumers on the system equilibrium strategy can also be analyzed.

The process of the parameters design and numerical value setting in this article is as follows. Firstly, the parameters and value settings of the game model were referred to the literatures Tang et al. [41] and Wu et al. [42], and the initial simulation value of the parameters were assigned. After consulting seven persons including academic experts and platform managers, set the value of variable parameters under different intensity, such as high and low, and values range of variable parameters of the influence of online public opinion caused by rights protection, airlines’ control on air ticket sales resources, obtained by consumers for rights protection. Finally, based on the news and reports of actual cases on OTA platforms such as Ctrip and Qunar, and considering the constraints that the parameters need to meet in the case 3 equilibrium analysis, the parameters and simulation value were determined. The initial simulation value of parameters is as follows:

$$\eta =0.1,\;F=200,\;\alpha =0.2,\;m=2,\;\lambda =0.1,\;K=5,\;N=10,\;M=5,\;Q=15,\;V=5,\;R_{1}=50,\;R_{2}=6,\;C_{1}=25,\;C_{2}=4,\;C_{3}=0.1,\;H=10,\;L_{1}=20,\;L_{3}=5,\;W=2000.$$

**Fig 1:**
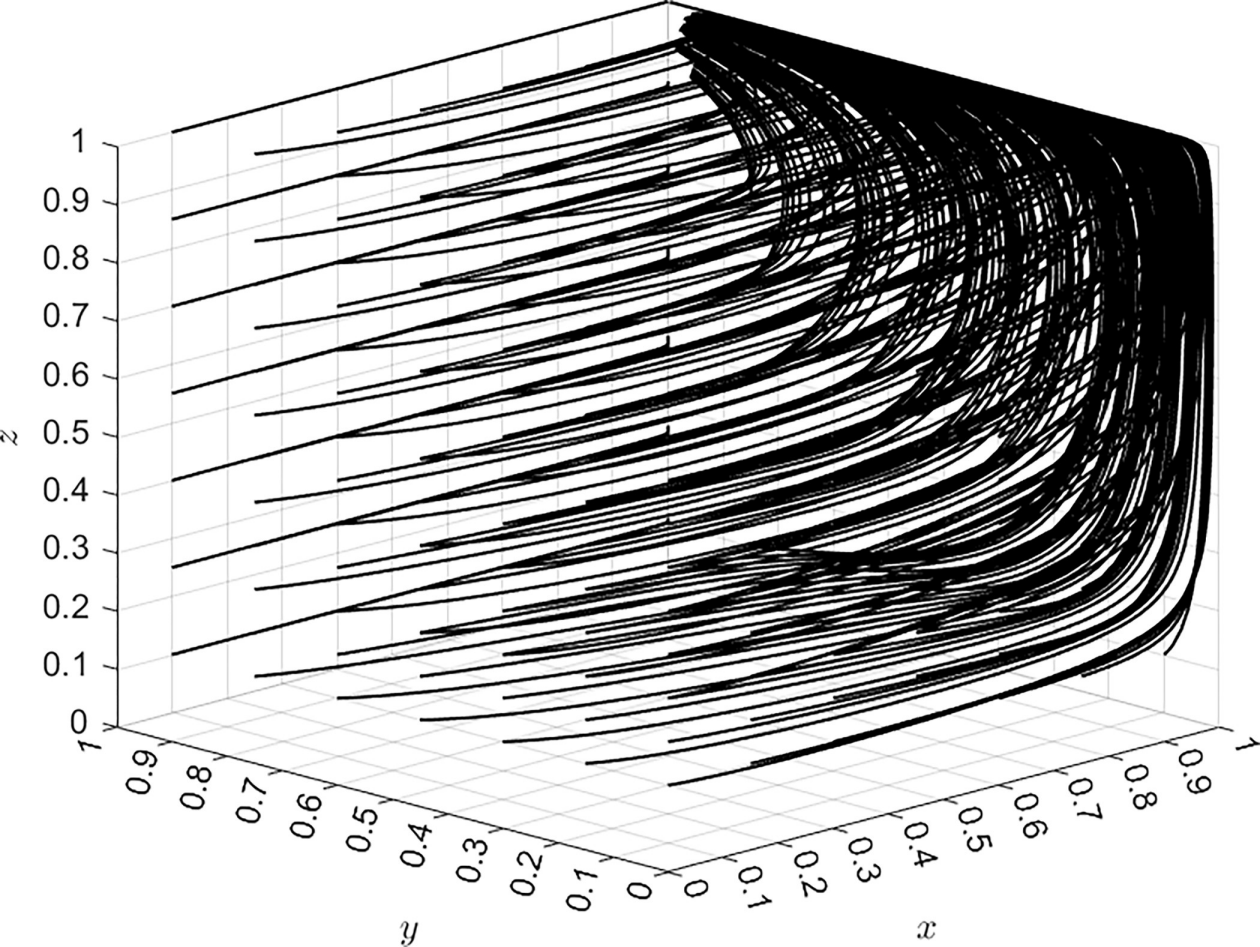
Evolution of system stability strategy.

The evolutionary result of the stable strategy of the three-party game system as a whole under Case C based on Matlab software is shown in Fig 1, where the simulation step of the probabilities of the initial strategy selection for three parties is 0.15. In Fig 1, the evolutionary dynamics of three value x, y, and z are also shown. x is the probability of airlines’ strategy selection, and the dynamics of x represents change trend of the airlines’ strategy probability over time when it is assigned an initial value of 0.2. y is OTA platforms’ strategy probability and z is the consumers’ probability. Their initial value is set as 0.2. As can be seen from Fig 1, after the probability of the three parties’ initial strategy selection is defined, the ESS of the system always tends to (positive regulation, compliant operation, rights protection) as time continues. The simulation result is used to verify the reasoning conclusion in Case C. Moreover, the greater the probability of initial strategy selection, the faster the system reaches the equilibrium state and the more significant the stability of the strategy. Case C is the optimal ESS. Fig 1 reflects the trend wherein the three-party strategy eventually evolves into the optimal strategy. The following simulation analysis is based on the simulation value of Fig 1 by changing the parameter values of different research variables. Analysis of the simulation results reveals the changes of these key parameters that impact the evolution of the system with respect to the optimal stability strategy. Generally, the probabilities of the initial strategy selection for three parties are *x* = 0.2, *y* = 0.2, and *z* = 0.2.

### Control degree of ticket sales resources

**Fig 2:**
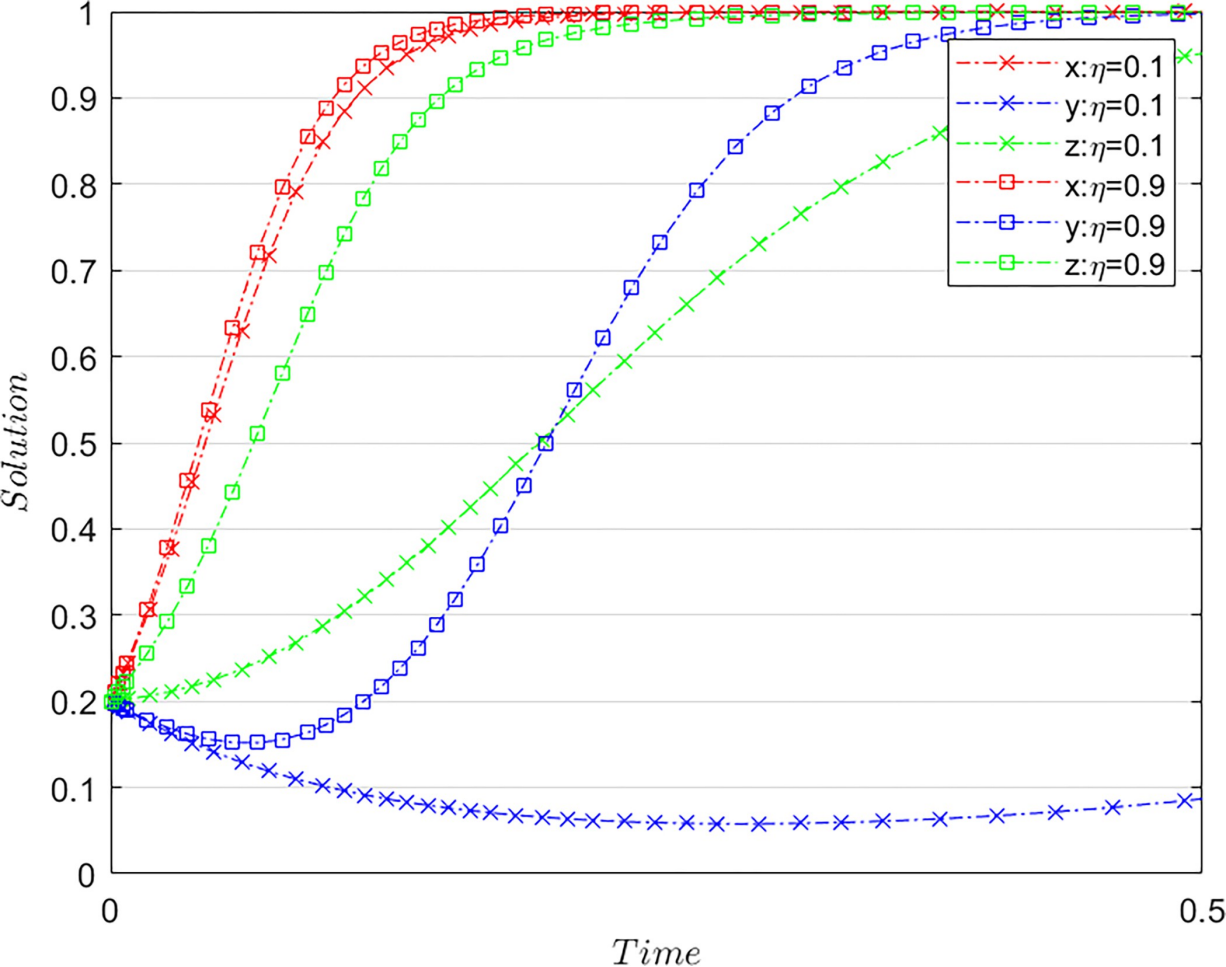
Influence of airlines’ control over sales resources on the system evolution.

Subject to the constraints of Case C, and meeting the basic assumptions based on the initial values of Fig 1 and maintaining the values of the other parameters, the value of *η* is altered. Specifically, we consider *η* = 0.1 and *η* = 0.9, which represent the different degrees of control airlines exert over ticket sales resources. The results of the system evolution paths are shown in Fig 2: The initial value *η* = 0.1 is depicted with a line marked by × and the comparative value *η* = 0.9 is shown with a line marked by ◻. The three main parties—airlines, OTA platforms, and consumers—are denoted by red, blue, and green lines, respectively. In the subsequent simulation result diagrams shown in Fig 2, the ratio, initial value, and three main bodies are distinguished using the same display method.

Clearly, improving airlines’ control over air ticket sales resources promotes the evolution of the system toward a stable state. When airlines’ control over sales resources gradually increases, the speed of OTA platforms toward non-misconduct continues to increase. This shows that if the condition of Case C is met, the increase in the degree of control of airlines’ sales resources prompts OTA platforms to adopt proper behavior. A high degree of control ensures that airlines have full initiative and voice in the control process. Thus, deeper control degree of airlines’ sales resources weakens the original network traffic advantage of OTA platforms, significantly suppressing the misconduct of platforms. Airlines’ degree of control also affects the strategy evolution speed of each airline and consumer; increasing airlines’ degree of control over sales resources enhances airlines’ enthusiasm for control and consumer rights protection. In 2015, after China’s state-owned assets supervision and administration commission required airlines to “increase the proportion of direct sales and reduce the proportion of agency sales,” airlines increased control over terminal sales resources by strengthening direct sales channels while tightening OTA platform management. For example, airlines directly suspended the cooperative relationship with Qunar, forcing it to stop using the pangolin in exchange for normalizing the cooperative relationship with airlines, thus avoiding further damage to the Qunar platform, airline revenue management, and travel interests of consumers.

### Internet public opinion

**Fig 3:**
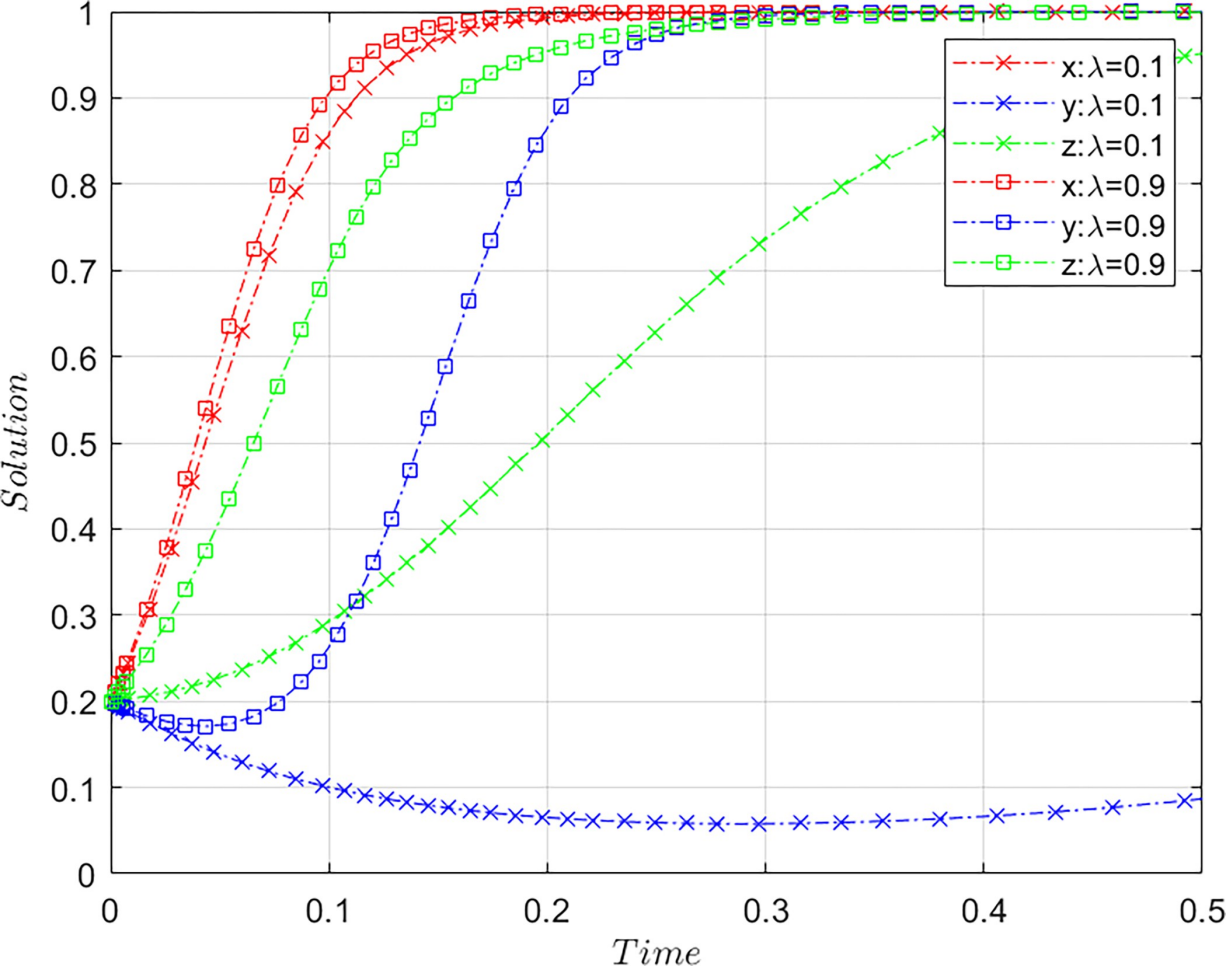
Influence of the degree of online public opinion on the system evolution.

Subject to the constraints of Case C, the value of *λ* is altered by meeting the basic assumptions based on the initial values of Fig 1 and maintaining the values of the other parameter. Specifically, we consider *λ* = 0.1 and *λ* = 0.9. *λ* = 0.1, set to its initial value, and *λ* = 0.9, set to its comparative value; together, they represent different types of online public opinion triggered by consumers rights protection. The results of the system evolution paths are shown in Fig 3.

Fig 3 reveals that the greater the impact of online public opinion, the more conducive it is for the system to remain in a stable state. When the impact of online public opinion is on the rise, airlines will accelerate to regulate positively, indicating that the increase in the impact of online public opinion brings more pressure on airlines. Consumers will have a negative impression of the airline because of its negligence as the scandal spreads; therefore, the airline is more likely to adopt a strategy of positive regulation to avoid damaging its image. Furthermore, OTA platforms accelerate the adoption of the non-misconduct strategy, as online public opinion is influenced by the rights protection of consumers, indicating that the evolution of the platform’s strategy is influenced by public opinion on the Internet and that the OTA platform faces, if any, the risk of reputational loss and economic loss caused by the intensely negative online public opinion. Therefore, to maintain its image and profits, the OTA platform would enhance its self-regulation. Hence, intense online public opinion curbs misconduct on OTA platforms.

Actor Han Xue revealed on Weibo her experience with bundle sales when she had bought an air ticket through Ctrip [43]. As a number of actors, and other key opinion leaders reposted her post, the number of reposts and likes mounted to over 100,000. *People’s Daily*, along with other news agencies, followed up on this incident, generating widespread concern. Finally, this controversy led to the rectification of the bundle sales of tickets carried out by Ctrip. This finding indicates that online public opinion plays an important role in platform regulation.

### Upper limit of the fine

**Fig 4:**
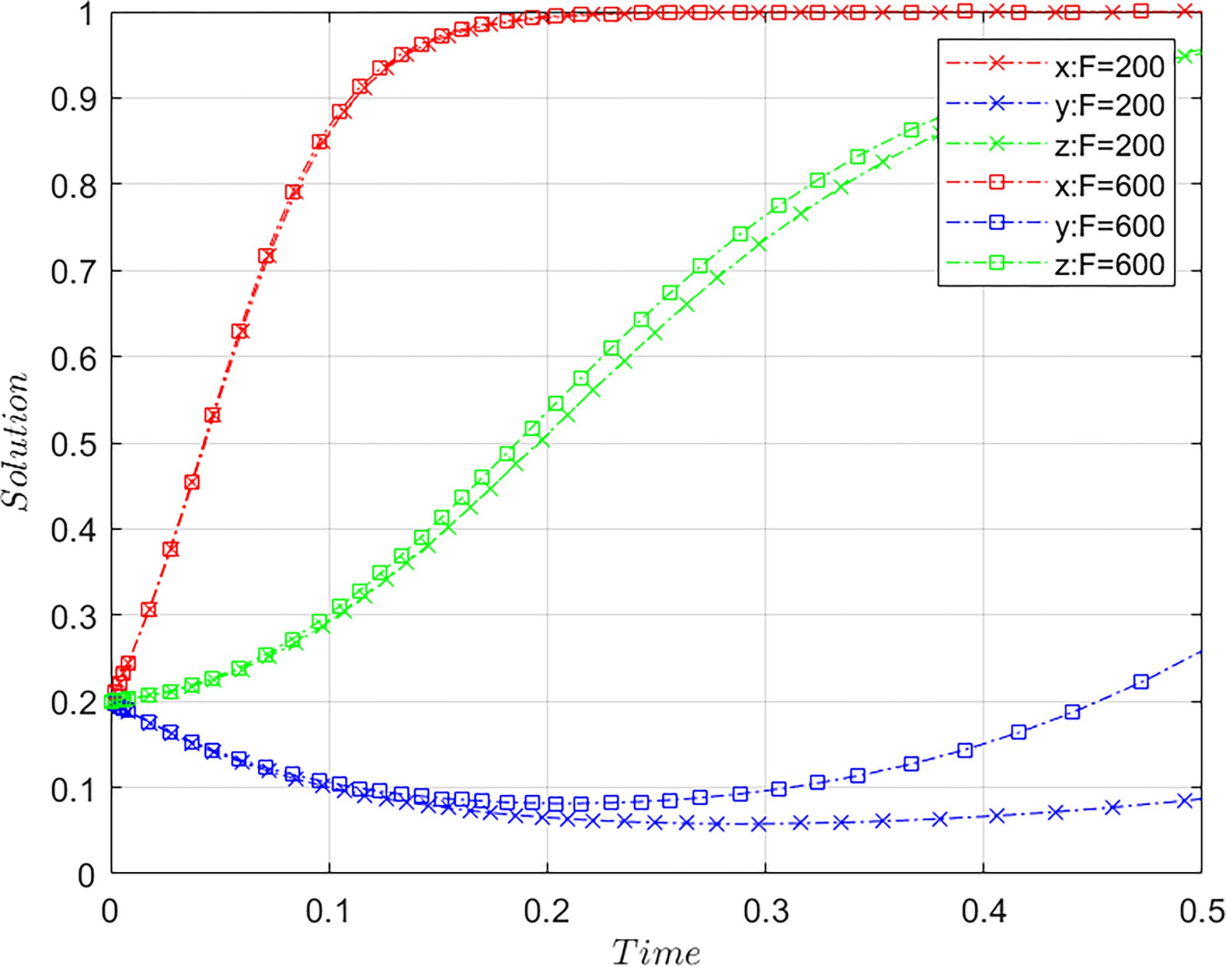
Influence of the upper limit of fine on system evolution.

Subject to the constraints of Case C, meeting the basic assumptions based on the initial values of Fig 1 and maintaining the values of the other parameters, the value of *F* is altered. Let *F* = 200 and *F* = 600. *F* = 200 is the initial value and *F* = 600 is the comparative value, representing the different upper limits of fines imposed on platforms by the airlines. The results of the system evolution paths are shown in Fig 4.

As the upper limit of the airlines’ fines increases, the time required for the three-party system to converge to a stable state gradually decreases. This implies that raising the upper limit of the fines is conducive to the evolution of the system toward stable strategies. Increasing the fine upper limit has a significant positive impact on the OTA platforms’ strategy evolution. When faced with higher fine limits imposed by airlines on misconduct, platforms tend to transition to a compliant operational strategy more rapidly. This suggests that elevating fines can effectively curb platform misconduct. However, merely increasing fines has relatively little impact on promoting collaborative governance between airlines and consumers.

### Combined effect of variable *F*, *η*, and *λ*

**Fig 5:**
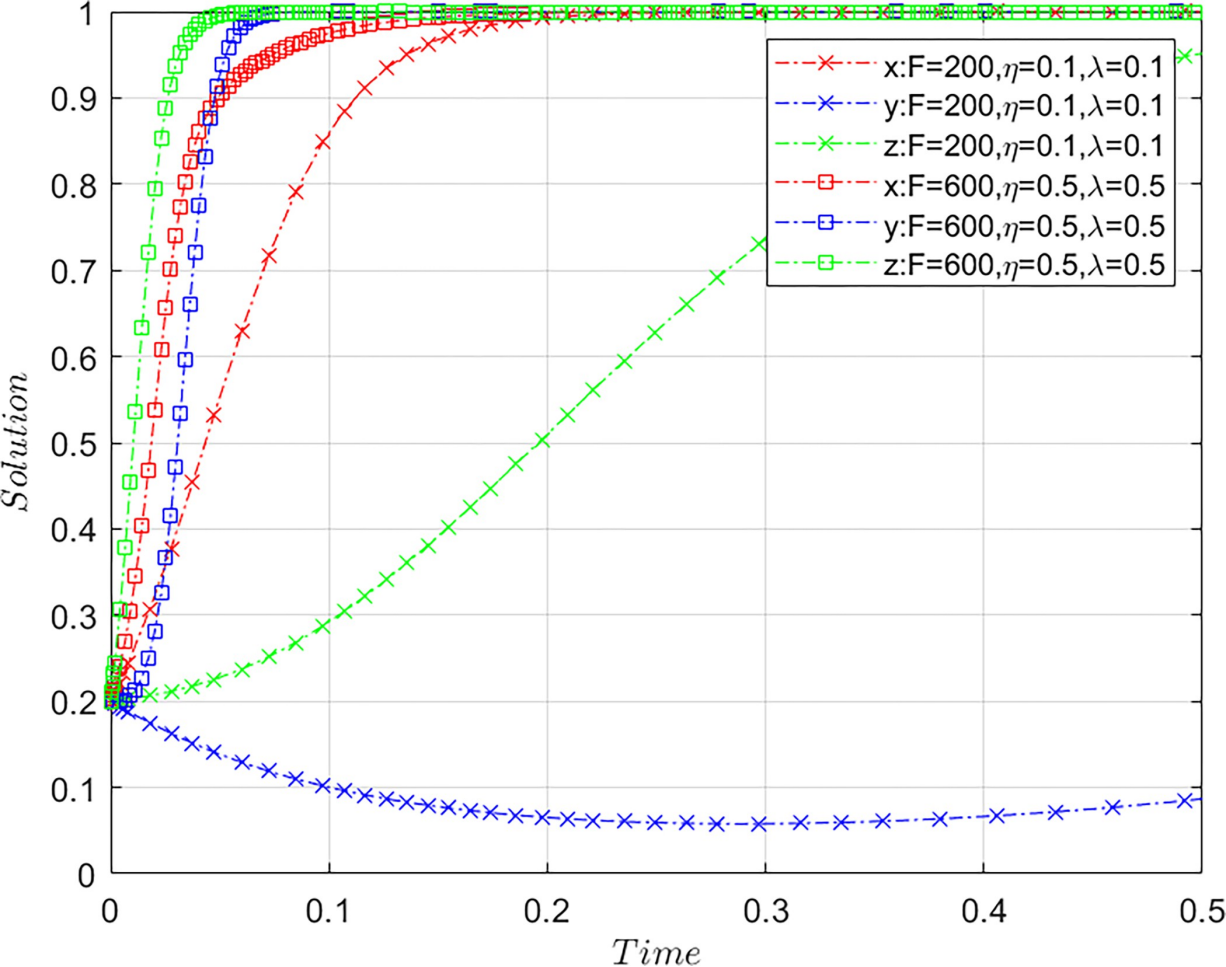
Synergy of the combination of *F*, *η*, and *λ*.

Subject to the constraints of Case C, the values of *F*, *η*, *λ* are altered together by meeting the basic assumptions based on the initial values of Fig 1 and maintaining the values of other parameters. Let *F* = 200, *η* = 0.1, *λ* = 0.1 and *F* = 600, *η* = 0.5, *λ* = 0.5, where *F* = 200, *η* = 0.1, *λ* = 0.1 are the initial values, while *F* = 600, *η* = 0.5, *λ* = 0.5 are comparative ones, representing different strategy groups of *F*, *η*, and *λ* with other parameters staying the same. The results of the system evolution paths are shown in Fig 5.

Evidently, as the upper limit of airlines’ fines increases and the airlines’ control over ticket sales resources and the influence of online public opinion, the time required for the system to converge to a stable state noticeably shortens. Compared with merely lifting the upper limit of fines in Fig 4, raising the upper limit of fines, together with enhancing control over ticket sales resources and strengthening online public opinion, is not only conducive to the strategy evolution of OTA platforms but also has a relatively greater positive influence on the evolution paths of airlines and consumers. Thus, merely increasing the upper limit of fines results in a relatively limited motivation for both airlines and consumers. To enhance effectiveness, this strategy should be combined with measures such as a high upper limit on fines, strong control over ticket sales resources, and influential online public opinion. These combined measures can more effectively encourage airlines to actively regulate platforms and prompt consumers to assert their rights, consequently ensuring that platform misconduct is effectively curtailed.

### Compensation coefficient

**Fig 6:**
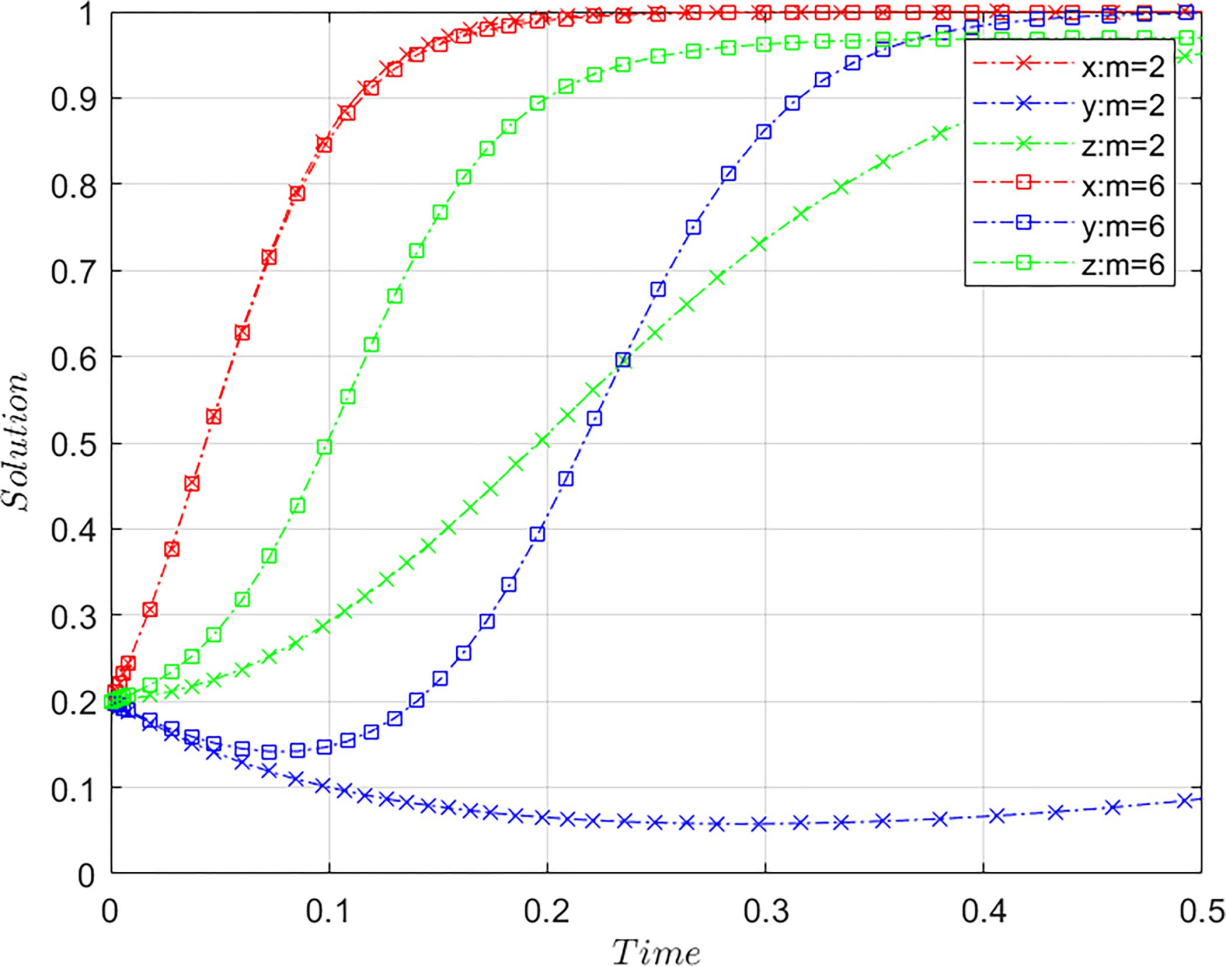
Influence of the compensation coefficient of platforms for consumers’ losses on the system evolution.

Subject to the constraints of Case C, the value of *m* was altered by meeting the basic assumptions based on the initial values of Fig 1 and with other parameter values being the same. Let *m* = 2 and *m* = 6, where *m* = 2 is the initial value and *m* = 6 is a comparative value, representing different coefficients of the compensation for consumers’ loss by the platforms. The results of the system evolution paths are shown in Fig 6.

The higher the compensation coefficient of platforms, the easier it is for the system to stabilize, indicating that an increase in the compensation coefficient of platforms promotes the system to achieve stability faster. The greater the compensation coefficient of platforms for consumers, the faster the convergence speed of platforms to a non-misconducting strategy. The relatively large compensation coefficient, which means more compensation OTA platforms need to pay to consumers, can apply high levels of economic pressure on platforms and curb misconduct effectively to make the OTA platform assume its social responsibility and operate legally. The convergence speed of consumers’ choices regarding the strategy of asserting their rights accelerates noticeably with an increase in the platforms’ compensation coefficient. This indicates that a higher compensation coefficient enhances the actual benefits for consumers who assert their rights, and offsets the costs they incur in protecting their rights. This approach encourages consumer engagement in collaborative governance by airlines for platform misconduct by encouraging them to actively participate.

### Compliant operation cost

**Fig 7:**
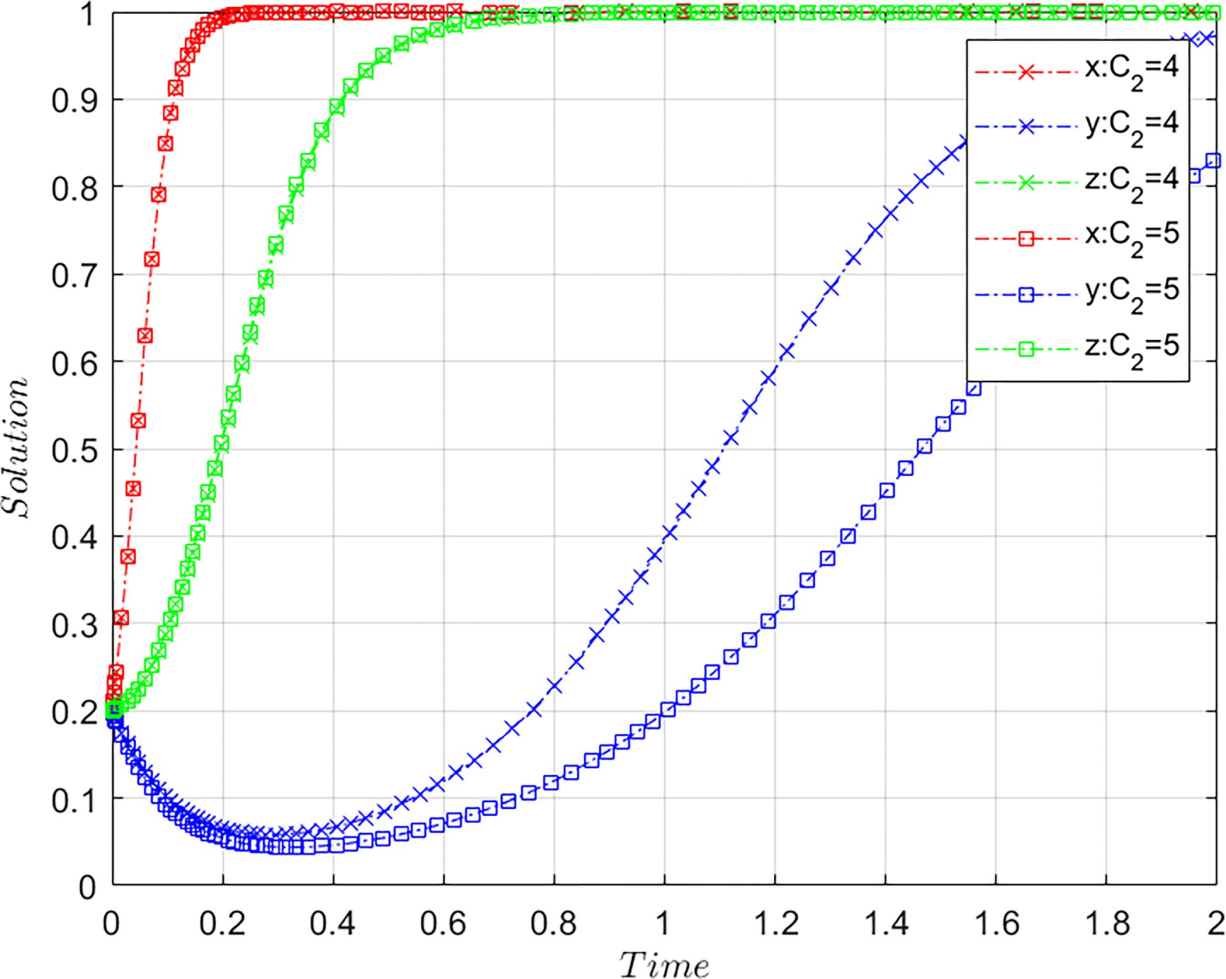
Influence of compliant operation cost of the platforms on system evolution paths.

Subject to the constraints of Case C, the values of *C*_*2*_ are altered by meeting the basic assumptions, based on the initial values of Fig 1, and maintaining the values of other parameters. Let *C*_2_ = 4 and C_2_ = 5, where *C*_2_ = 4 are initial values and C_2_ = 5 comparative values, and *C*_2_ represents the additional costs incurred by the platforms during compliance operations. The results of system evolution paths are illustrated in Fig 7.

When the additional costs of compliant operation of the platform increases, the speed of the platform’s tendency towards compliant operation strategies will significantly slow down. Therefore, although the evolutionary trend of the airlines and consumers toward the (1,1) state remains unchanged, the pace at which the three-party system as a whole converges toward the state of (1,1,1) noticeably slows. This implies that the high compliant operation cost inhibits the actualization of a stable system state. At the same time, it also reflects that the platforms have a high sensitivity to additional operating costs, and the increased operating costs associated with compliant operations will drive the platforms’ adverse selection.

### Benefits from misconduct

**Fig 8:**
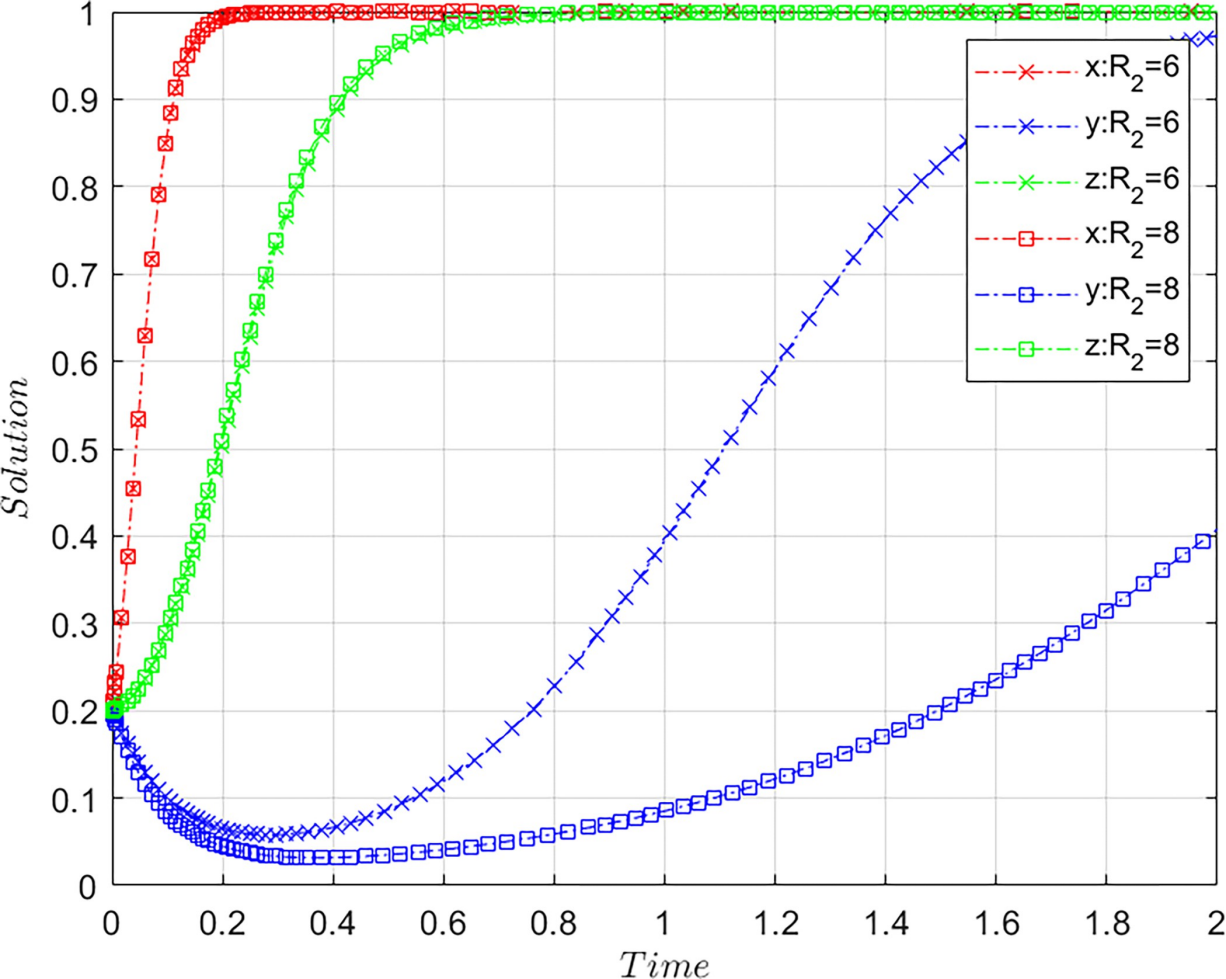
Influence of the platforms’ benefits from misconduct on system evolution paths.

Under the constraints of Case C, the values of *R*_*2*_ are altered while satisfying the basic assumptions, based on the initial values of Fig 1, and maintaining the values of other parameters. Let *R*_2_ = 6 and *R*_2_ = 8, where *R*_2_ = 6 are initial values and *R*_2_ = 8 comparative values, and *R*_2_ represents the platforms’ benefits from misconduct, with other parameters being the same. The paths of system evolution are showed in Fig 8.

The greater the incremental gains obtained from misconduct for OTA platforms compared with compliant operations, the slower the convergence of the platforms’ choices toward compliant behaviors. Although the evolutionary trend of the airlines and consumers toward the (1,1) state remains unchanged, the pace at which the three-party system as a whole converges toward the state of (1,1,1) noticeably slows. This implies that the platforms’ benefits from the misconduct inhibit the actualization of a stable system state. Benefits from misconduct greatly affect platform choice, and high benefits stimulate opportunistic behaviors of the platform. Reducing the income from misconduct helps restrain the platforms’ opportunism.

### Cost for rights protection

**Fig 9:**
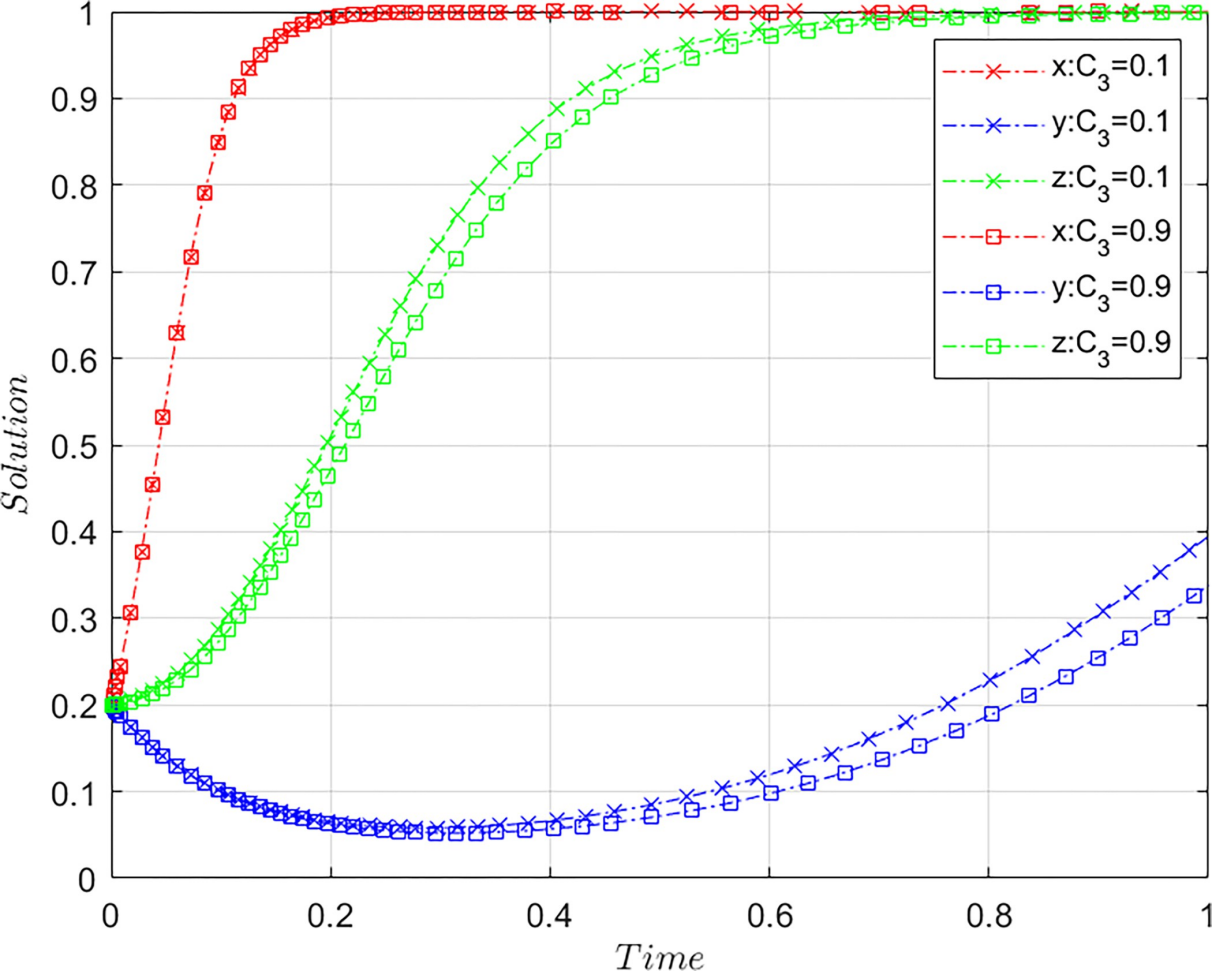
Influence of consumers’ rights protection cost on the system evolution.

Subject to the constraints of Case C, the value of *C*_*3*_ is altered by meeting the basic assumptions, based on the initial values of Fig 1, and maintaining the values of the other parameters. Let *C*_3_ = 0.1 and *C*_3_ = 0.9, where *C*_3_ = 0.1 is the initial value and *C*_3_ = 0.9 is a comparative value, representing different costs of consumers rights protection, with other parameters being the same. The results of the system evolution paths are shown in Fig 9.

Evidently, with a gradual increase in customers’ cost for rights protection, the speed of the system tending to a stable state gradually slows down, indicating that the cost of consumer rights protection hinders the realization of the stable state of the system. As the cost of rights protection increases, the convergence speed of consumers’ choice of rights protection strategies slows significantly, indicating that consumers’ strategic choices are more sensitive to the cost of rights protection, and a higher cost will dampen consumers’ enthusiasm for rights protection. Reducing the cost of rights protection can significantly weaken consumers’ resistance to rights protection, prompting them to take measures against the platform’s non-compliant behavior and protect their legitimate rights and interests. In addition, when the cost of rights protection increases, the speed of OTA platforms tending toward non-misconduct strategies also gradually slows, indicating that a higher cost of consumers rights protection will breed the non-compliant behavior of the OTA platform. Therefore, reducing the cost of rights protection motivates consumers to actively protect their rights and weakens the platform’s vested interests, thereby encouraging OTA platforms to choose a complaint operation strategy. According to the Xinhua News Agency, a consumer who purchased a ticket at Tuniu in 2019 but did not use it and was unable to obtain a refund after investing two years in rights protection shows that the high cost of rights protection has created a serious obstacle for consumers in protecting their rights [44]. Issues such as the inability to fully refund tickets, prolonged delays in refunds, fees of up to 80% for refunds, and resting the burden on airlines make it difficult for consumers to protect their rights [7, 45]. Therefore, it is necessary to create a favorable environment for consumers to actively protect their rights and participate in platform governance.

## Conclusion

To solve the problem of OTA platforms’ misconduct in ticket sales, we build an evolutionary game model with airlines, OTA platforms, and consumers as the main parties and consider the choice of consumer engagement strategies under airlines’ different incentives. Using Matlab to simulate the stability strategy of a tripartite game system, we also discuss the influence of key parameters such as airlines’ control over ticket sales resources and the impact of online public opinion on system evolution. The main conclusions and suggestions are as follows:

First, airlines’ strategic choices are influenced mainly by the degree of control over air ticket sales resources, upper limit of fines on the platforms, and influence of online public opinion. Airlines should increase the proportion of direct sales and reduce the proportion of agency sales, that is, enhance air companies’ control over sales channels to make them less dependent on OTA platforms, thus curbing the platform’s monopoly on the customer base. Official websites must sell airline tickets to optimize online services and become more user-friendly, including providing website guidance, website maps, keyword searches, and internal and external links. To reduce the lock-in effect of OTA platforms on airlines and consumers, airlines should utilize the airport and onboard facilities for offline marketing. They should publicize the official app so as to attract more consumers to purchase air tickets through official online channels, thus increasing the customer stickiness of the airline channel as well as the ratio of tickets that are directly sold. Simultaneously, the upper limit of the fine for platforms engaging in misconduct should be raised, that is, enhancing the punishment intensity on the platforms, to ensure airlines’ effective intervention for the benefit of the platforms. In addition, airlines should guide consumers to use online public opinion reasonably, urge platforms to actively deal with consumer complaints, and promptly control negative public opinion within a certain range to avoid negative impacts on the airlines and platforms.

Second, strategy selection of OTA platforms is significantly influenced by the additional operating cost when platforms are compliant, increased revenue when there is misconduct, the compensation coefficient, and the degree of online public opinion. The higher the revenue from misconduct, the higher the costs to comply, so the weaker their motivation for compliant operation, the less likely the OTA is to comply. Moreover, the greater the platform’s compensation coefficient to the consumer and the impact of rights protection on online public opinion, the greater the loss caused by misconduct, and the more effective the curbing of the misconduct of OTA platforms will be. The pressure of public opinion and reputation loss increase the operating costs of platforms, which is consistent with previous research perspectives [26]. Market regulators should take action to increase the cost of platforms out of compliance, such as intensifying the law enforcement of anti-unfair competition and price supervision, establishing and improving the information-sharing mechanism, improving the efficiency of capturing and feedback on misconduct clues, and enhancing the monitoring level of complaints and key public opinions. Airlines should reward platforms that act properly by granting priority service permissions, such as front seat booking and airport priority passage, to assist platforms in attracting customers, thus picking up the return of OTA platforms. It is also beneficial to introduce the third-party financial support for delayed insurance and carry out ticketing resource tilt and commission rewards, thereby partially decreasing the costs of the platforms in compliance.

Third, the cost of rights protection is the focus of consumer strategy selection. The lower the cost of rights protection is, the more consumers are inclined to choose a rights protection strategy. The cost of rights protection hinders consumer rights protection, and the high cost of rights protection is not conducive to consumers implementing rights protection measures. The active participation of consumers is vital to correcting market failure, which corresponds to the research viewpoints of others [46]. Reducing the cost of rights protection can further enhance consumer willingness to protect their rights. In view of the fact that important data or information are under the absolute control of platforms, consumers in a weak position often need to spend a lot of time, manpower, and expenses providing evidence of infringement. Therefore, it is necessary for airlines to achieve greater success in helping consumers. For example, airlines can set up special rights protection channels for OTA ticket sales, encourage platform users to provide clues about infringement, reward whistleblowers, urge platforms to cooperate in verification, and implement compensation measures to protect consumers’ rights and interests. Simultaneously, airlines should also improve their own treatment systems for consumer rights protection, increase the speed of response, and promptly verify and respond to consumer rights protection information to provide consumers with a convenient and efficient environment for rights protection.

In summary, collaborative governance is required to suppress platform misconduct, and this finding supports previous views [18, 22, 25, 26]. What sets our findings apart is that our study considers the synergy between airlines and consumers. For the gaming system as a whole, the combined effect of various measures, such as airlines’ strict control over ticket sales resources, high fines, and strong online public opinion, is better than a single measure for increasing the level of fines. Increasing the degree of airline control over ticket sales resources and the upper limit of airline fines on the platform can promote the overall evolution of the system in a positive direction. A high degree of control over sales resources and the upper limit of fines will make the misconduct platforms face a high level of punishment, and the active airlines and consumers will gain more benefits, which will enhance the willingness of the airlines and consumers to co-manage the platforms and inhibit their misconduct. While improving the control of sales resources and the upper limit of fines, the media should reasonably direct the influence of online public opinion created by consumers’ legitimate rights protection, put pressure on inactive airlines and platforms with misconduct, and weaken their reputations and image effects. Therefore, to promote the airlines to actively supervise the platform and force the platforms to strengthen self-regulation, it is also helpful to encourage consumers to increase the dissemination of public opinion through multi-media, multichannel legal voice and other methods, and exert enough influence on the airlines with pressing public opinions. Simultaneously, it is necessary to actively improve the channels for consumers to protect their legitimate rights and coordinate the increase in the level of platform penalties with measures to reduce the cost of consumer rights protection so as to increase the level of benefits for consumer, strengthening consumers’ willingness to participate in the governance of platform misconduct and finally forming platform self-regulation based on market mechanisms under the collaborative governance of airlines and consumers, so as to effectively prevent the OTAs platforms’ opportunistic behavior.

There are still some extensions for future research that are neglected for the sake of simplicity of the model and analysis. This study assumes that the choice between consumer participation and airline supervision is not influenced by government policy, although the actual situation may be more complicated. The model did not consider the degree of the diffusion of online public opinion, although it may have had a different impact. Major extension work is also needed, that is, the game dynamics between non-low-cost carriers and OTA. This study focuses on analyzing the behavior of non-low-cost airlines. LCCs have a higher market penetration rate in the low value-end consumers with price-sensitive, whereas non-LCCs have a higher markets penetration rate in middle value-end and high value-end consumers. The latter is more dependent on customer drainage from the platforms. There is a love-and-kill relationship between airlines and OTA platforms. However, there are differences between LCCs and non-LCCs in terms of dependence on the platforms, the control force of the platforms, and the control of ticket resources. Non-LCCs can reduce their dependence on platforms through measures such as the frequent flyer program, and platforms often compromise in the face of threats from non-LCCs. However, LCCs rely heavily on platforms and have limited control. The situation of the LCCs’ game relationship with the platforms is different from that in this study, and the results of the game analysis may also be different. Three extensions can be implemented in future.
